# A new mononuclear neutral high-spin iron(III) complex with the different tridentate ligands 5-bromo­salicyl­aldehyde (pyridin-2-yl)hydrazone and 5-bromo­salicyl­aldehyde thio­semicarbazone

**DOI:** 10.1107/S2056989018001263

**Published:** 2018-01-31

**Authors:** Yun Zhao, Xiao-Feng Shen, Li-Fang Zhang

**Affiliations:** a School of Chemical Engineering and Technology, China University of Mining and Technology, Xuzhou 221116, People’s Republic of China

**Keywords:** iron(III) complex, 5-bromo-salicyl­aldehyde-2-pyridyl­hydrazone, 5-bromo-salicyl­aldehyde-thio­semicarbazone, high-spin, magnetic property, crystal structure

## Abstract

The title neutral mononuclear complex, [1-(5-bromo-2-oxido­benzyl­idene)thio­semicarbazidato](4-bromo-2-{[2-(pyridin-2-yl)hydrazinyl­idene]meth­yl}pheno­lato)iron(III), [Fe(C_8_H_6_BrN_3_OS)(C_12_H_9_BrN_3_O)] (I), crystallizes in the monoclinic space group *C*_2_/*c* and has two different planar tridentate ligands. The central Fe^III^ ion is coordinated to three N, two O and one S atom, forming a distorted octa­hedral FeN_3_O_2_S coordination geometry. In the crystal, the complex mol­ecules are linked by N—H⋯O and N—H⋯N hydrogen bonds and π–π inter­actions into layers parallel to (100). Magnetic measurements show that the central Fe^III^ ion is in the high-spin state; this is also supported by the bond distances around the Fe^III^ ion.

## Abstract 2

The mol­ecular and crystal structure of a new mononuclear neutral high-spin iron(III) complex with two different tridentate ligands has been studied by an X-ray diffraction analysis.

## Chemical context   

Much attention have been paid to the design and synthesis of Fe^III^ complexes for magnetic materials owing to their inter­esting thermal- or light-induced spin conversion between the high-spin (HS, *S* = 5/2) and low-spin (LS, *S* = 1/2) states (Li *et al.*, 2013 ▸; Phonsri *et al.*, 2017 ▸; Sato *et al.*, 2007 ▸). It is well known that the organic ligands usually play a significant role in the crystal structures and magnetic properties of metal complexes (Ni *et al.*, 2017 ▸; Zhang *et al.*, 2016 ▸). Up to date, many Fe^III^ complexes with spin-crossover (SCO) behavior have been designed and synthesized through the subtle design and combination of different ligands. Among the many organic ligands, Schiff bases are the most common ligands for new Fe^III^ complexes due to their convenient synthesis and regulation. Compared with homo-ligand complexes, the employment of mixed ligands provides more selection and modification strategies for new magnetic complexes. In previous reports, the ligands 5-bromo-salicyl­aldehyde-2-pyridyl­hydrazone (5-Br-Hpsal), 5-bromo-salicyl­aldehyde-thio­semicarbazone (5-Br-H_2_thsa) and their derivatives have been explored to assembly Fe^III^ and Mn^III^ complexes with SCO behavior (Shongwe *et al.*, 2014 ▸). Recently, we obtained the title complex, [(C_20_H_15_N_6_O_2_SBr_2_)Fe] (I), using 5-Br-Hpsal and 5-Br-H_2_thsa ligands. Herein, we report the crystal structure and magnetic property of this iron(III) complex.

## Structural commentary   

**Figure 1:**
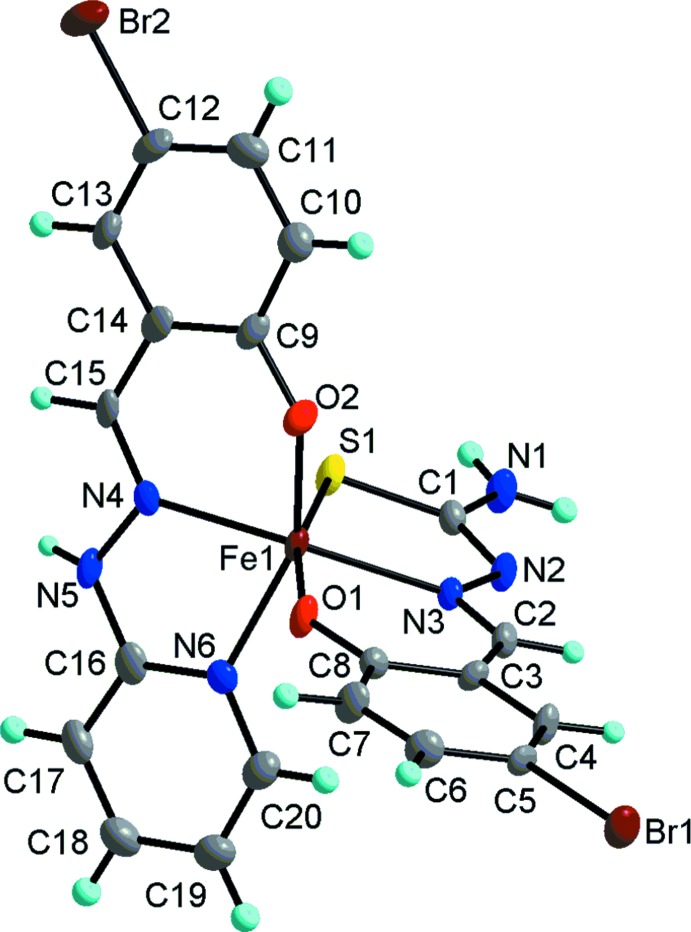
Mol­ecular structure of (I) with 30% probability displacement ellipsoids.

The title complex (Fig. 1 ▸) crystallizes in the monoclinic space group *C*_2_/*c*. Compound (I) is a neutral mononuclear complex with two different rigid tridentate ligands – 5-Br-psal^−^ and 5-Br-thsa^2–^ – which adopt a meridional coordination mode. The central Fe^III^ ion lies almost within the plane of each ligand **[give deviations]** and is coordinated to three nitro­gen, two oxygen and one sulfur atoms from the two tridentate 5-Br-psal^−^ and 5-Br-thsa^2–^ ligands, forming a distored octa­hedral FeN_3_O_2_S geometry. The Fe—O bond lengths are 1.943 (3) and 1.931 (3) Å, the Fe—N bond lengths range from 2.142 (3) to 2.157 (3) Å, and the Fe1—S1 bond length is 2.4093 (14) Å. All the bond lengths are normal and agree well with those in related high-spin state Fe^III^ compounds (Li *et al.*, 2013 ▸; Phonsri *et al.*, 2017 ▸). The C1—S1 bond length [1.720 (4) Å] is comparable with the ordinary C—S bond length (Li & Sato, 2017 ▸), whereas the C1=N2 and C2=N3 bond distances [1.314 (5) and 1.287 (5) Å, respectively] are significantly smaller than those of C1—N1 [1.350 (5) Å] and C16—N5 [1.377 (6) Å] indicating the double-bond character. The bond angles further evidence the significantly distorted octa­hedral coordination geometry around the Fe^III^ ion.
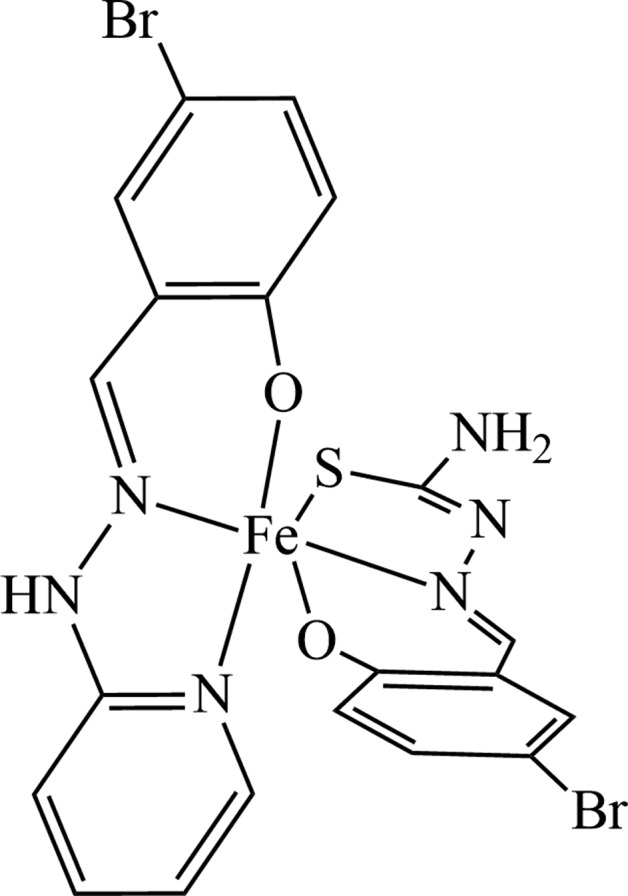


## Supra­molecular features   

**Figure 2:**
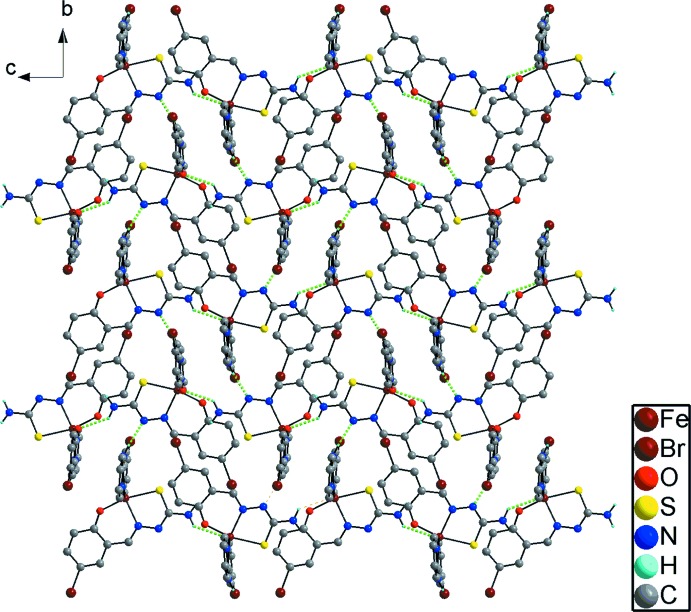
The layered structure of (I) formed through hydrogen bonds (green dotted lines) and π–π inter­actions.

In the crystal, there are two independent hydrogen bonds (Table 1 ▸), which link the complex mol­ecules into layers parallel to (100) (Fig. 2 ▸). In addition, there exist relatively strong π–π inter­actions between the pyridine and benzene rings of the 5-Br-psal^−^ ligands with a shortest inter­atomic distance of 3.485 (3) Å (Fig. 2 ▸).

## Magnetic properties   

**Figure 3:**
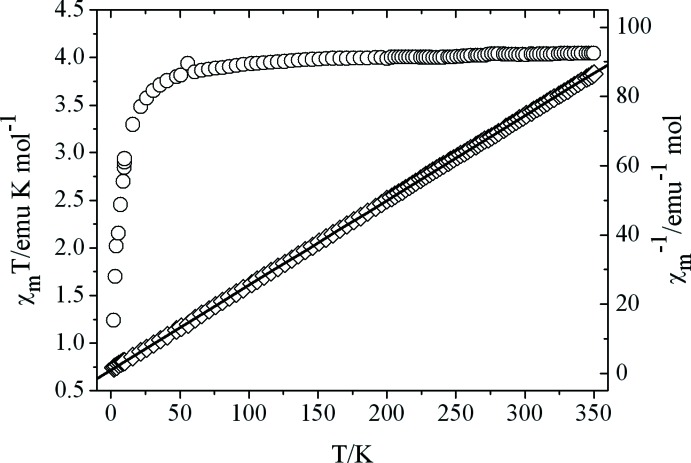
Temperature dependencies of χ_m_ *T* and χ_m_ *versus* temperature (T) for complex (I) measured under an applied field of 2000 Oe. The solid line represents the fitting curve based on the Curie–Weiss law.

The magnetic susceptibilities of (I) have been measured in the temperature range 2–350 K under an applied magnetic field strength of 2000 Oe by SQUID magnetometry. A plot of χ_m_ *T* versus *T* is presented in Fig. 3 ▸, where χ_m_ represents the molar magnetic susceptibility per Fe^III^ unit. The χ_m_ *T* value is 4.042 emu K mol^−1^ at room temperature, which is slightly smaller than the expected value of 4.375 emu K mol^−1^ for the single spin carrier of high-spin Fe^III^ (*S* = 5/2) based on *g* = 2.0. The measurement of the magnetic property shows that the Fe^III^ ion is in the high-spin state, which agrees well with the above-mentioned bond lengths around the Fe^III^ ion. The χ_m_ *T* value keeps nearly constant with decreasing temperature until around 75 K, and then it decreases quickly to a minimum value of 1.12 emu K mol^−1^ at 2.0 K. This tendency to change of the χ_m_ *T* curve indicates the existence of overall weak anti­ferromagnetic inter­actions in (I). The magnetic susceptibilities in the range of 2–350 K comply well with the Curie–Weiss law with a negative Weiss constant θ = −4.28 K, and Curie constant *C* = 4.08 emu K mol^−1^, which further confirms the presence of overall inter­molecular anti­ferromagnetic inter­actions between neighboring Fe^III^ ions through inter­molecular hydrogen bonds and π–π inter­actions in complex (I).

## Synthesis and crystallization   

All reactions were conducted in air using reagent grade solvents. The 5-Br-Hpsal and 5-Br-H_2_thsa ligands were synthesized by refluxing equimolar 5-bromo­salicyl­aldehyde with thio­semicarbazone and 2-pyridyl­hydrazine, respectively, in an ethanol solvent. All other chemicals were purchased from the Sigma Aldrich Chemical Company and used as received. The precursors [Fe(5-Br-psal)_2_]Cl and Li[Fe(5-Br-thsa)_2_] were prepared according to literature methods (Phonsri *et al.*, 2016 ▸). [Fe(5-Br-psal)_2_]Cl (0.2 mmol) and Li[Fe(5-Br-thsa)_2_] (0.2 mmol) were dissolved in aceto­nitrile (20 mL). The mixture was filtered and kept at room temperature for two days. Brown block-shaped single crystals were collected with a relatively high yield of 76%. Elemental analysis calculated for C_20_H_15_N_6_O_2_SBr_2_Fe: C, 38.80%; H, 2.44%; N, 13.57%; found: C, 38.72%, H, 2.38%; N, 13.62%.

## Refinement   

Crystal data, data collection and structure refinement details are summarized in Table 2 ▸. The amino-H atom of 5-Br-psal^−^ was found from the difference-Fourier map and refined isotropically. All other hydrogen atoms were placed in calculated positions with C—H = 0.88–0.95 Å and refined in the riding model with fixed isotropic displacement parameters [*U*_iso_(H) = 1.2*U*_eq_(C,N)].

## Supplementary Material

Crystal structure: contains datablock(s) I, global. DOI: 10.1107/S2056989018001263/kq2017sup1.cif

Structure factors: contains datablock(s) I. DOI: 10.1107/S2056989018001263/kq2017Isup2.hkl

CCDC reference: 1818280

Additional supporting information:  crystallographic information; 3D view; checkCIF report

## Figures and Tables

**Table 1:** Hydrogen-bond geometry (Å, °)

*D*—H⋯*A*	*D*—H	H⋯*A*	*D*⋯*A*	*D*—H⋯*A*
N5—H5⋯N2^i^	0.83 (4)	2.00 (4)	2.825 (5)	171 (4)
N1—H1*A*⋯O2^ii^	0.88	2.29	2.987 (4)	136

Symmetry codes: (i) 
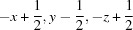
; (ii) 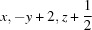.

**Table 2:** Experimental details

Crystal data	
Chemical formula	[Fe(C_8_H_6_BrN_3_OS)(C_12_H_9_BrN_3_O)]
*M*_r_	619.11
Crystal system, space group	Monoclinic, *C*2/*c*
Temperature (K)	123
*a*, *b*, *c* (Å)	21.145 (4), 14.738 (3), 15.471 (3)
β (°)	112.47 (3)
*V* (Å^3^)	4455.2 (18)
*Z*	8
Radiation type	Mo *K*α
μ (mm^−1^)	4.39
Crystal size (mm)	0.12 × 0.10 × 0.08

Data collection	
Diffractometer	Bruker APEXII CCD area-detector
Absorption correction	Multi-scan (*CrystalClear*; Rigaku, 2008 ▸)
*T*_min_, *T*_max_	0.576, 0.707
No. of measured, independent and observed [*I* > 2σ(*I*)] reflections	17937, 5012, 3514
*R*_int_	0.074
(sin θ/λ)_max_ (Å^−1^)	0.649

Refinement	
*R*[*F* ^2^ > 2σ(*F* ^2^)], *wR*(*F* ^2^), *S*	0.048, 0.106, 1.00
No. of reflections	5012
No. of parameters	293
H-atom treatment	H atoms treated by a mixture of independent and constrained refinement
Δρ_max_, Δρ_min_ (e Å^−3^)	0.53, −0.76

Computer programs: *CrystalClear* (Rigaku, 2008 ▸), *SHELXS97* and *SHELXTL* (Sheldrick, 2008 ▸), *SHELXL2014* (Sheldrick, 2015 ▸) and *publCIF* (Westrip, 2010 ▸).

## supplementary crystallographic information

### Crystal data

**Table:**

[Fe(C_8_H_6_BrN_3_OS)(C_12_H_9_BrN_3_O)]	*F*(000) = 2440
*M**_r_* = 619.11	*D*_x_ = 1.846 Mg m^−^^3^
Monoclinic, *C*2/*c*	Mo *K*α radiation, λ = 0.71073 Å
*a* = 21.145 (4) Å	Cell parameters from 2456 reflections
*b* = 14.738 (3) Å	θ = 3.0–26.6°
*c* = 15.471 (3) Å	µ = 4.39 mm^−^^1^
β = 112.47 (3)°	*T* = 123 K
*V* = 4455.2 (18) Å^3^	Block, brown
*Z* = 8	0.12 × 0.10 × 0.08 mm

### Data collection

**Table:**

Bruker APEXII CCD area-detector diffractometer	3514 reflections with *I* > 2σ(*I*)
Radiation source: Rotating Anode	*R*_int_ = 0.074
ω scans	θ_max_ = 27.5°, θ_min_ = 3.1°
Absorption correction: multi-scan (CrystalClear; Rigaku, 2008)	*h* = −27→25
*T*_min_ = 0.576, *T*_max_ = 0.707	*k* = −18→19
17937 measured reflections	*l* = −20→18
5012 independent reflections	

### Refinement

**Table:**

Refinement on *F*^2^	Primary atom site location: difference Fourier map
Least-squares matrix: full	Secondary atom site location: difference Fourier map
*R*[*F*^2^ > 2σ(*F*^2^)] = 0.048	Hydrogen site location: mixed
*wR*(*F*^2^) = 0.106	H atoms treated by a mixture of independent and constrained refinement
*S* = 1.00	*w* = 1/[σ^2^(*F*_o_^2^) + (0.0455*P*)^2^ + 0.2543*P*] where *P* = (*F*_o_^2^ + 2*F*_c_^2^)/3
5012 reflections	(Δ/σ)_max_ = 0.001
293 parameters	Δρ_max_ = 0.53 e Å^−^^3^
0 restraints	Δρ_min_ = −0.76 e Å^−^^3^

### Special details

**Table:**

Geometry. All esds (except the esd in the dihedral angle between two l.s. planes) are estimated using the full covariance matrix. The cell esds are taken into account individually in the estimation of esds in distances, angles and torsion angles; correlations between esds in cell parameters are only used when they are defined by crystal symmetry. An approximate (isotropic) treatment of cell esds is used for estimating esds involving l.s. planes.

### Fractional atomic coordinates and isotropic or equivalent isotropic displacement parameters (Å^2^)

**Table:**

	*x*	*y*	*z*	*U*_iso_*/*U*_eq_	
Fe1	0.25435 (3)	0.91027 (4)	0.12510 (4)	0.01599 (15)	
Br1	0.11616 (2)	1.34761 (3)	−0.13350 (3)	0.02748 (14)	
Br2	0.56554 (3)	0.64748 (4)	0.14892 (4)	0.04080 (16)	
O1	0.20626 (15)	0.96909 (19)	0.00536 (18)	0.0228 (7)	
O2	0.33707 (15)	0.91116 (19)	0.1001 (2)	0.0226 (7)	
S1	0.30696 (6)	0.87380 (7)	0.28908 (7)	0.0232 (3)	
N1	0.32499 (18)	0.9869 (2)	0.4275 (2)	0.0250 (8)	
H1A	0.3225	1.0394	0.4532	0.030*	
H1B	0.3446	0.9402	0.4631	0.030*	
N2	0.27026 (18)	1.0507 (2)	0.2850 (2)	0.0184 (8)	
N3	0.24650 (17)	1.0386 (2)	0.1873 (2)	0.0160 (7)	
N4	0.26090 (17)	0.7660 (2)	0.1140 (2)	0.0165 (7)	
N5	0.20634 (19)	0.7168 (2)	0.1166 (2)	0.0212 (8)	
N6	0.15575 (18)	0.8575 (2)	0.1092 (2)	0.0198 (8)	
C1	0.2987 (2)	0.9781 (3)	0.3335 (3)	0.0183 (9)	
C2	0.2241 (2)	1.1126 (3)	0.1416 (3)	0.0191 (9)	
H2	0.2272	1.1656	0.1780	0.023*	
C3	0.1945 (2)	1.1242 (3)	0.0406 (3)	0.0171 (9)	
C4	0.1734 (2)	1.2118 (3)	0.0059 (3)	0.0190 (9)	
H4	0.1792	1.2605	0.0484	0.023*	
C5	0.1448 (2)	1.2278 (3)	−0.0876 (3)	0.0182 (9)	
C6	0.1360 (2)	1.1570 (3)	−0.1512 (3)	0.0252 (10)	
H6	0.1161	1.1686	−0.2165	0.030*	
C7	0.1558 (2)	1.0713 (3)	−0.1194 (3)	0.0246 (10)	
H7	0.1490	1.0235	−0.1632	0.030*	
C8	0.1863 (2)	1.0516 (3)	−0.0228 (3)	0.0170 (9)	
C9	0.3869 (2)	0.8521 (3)	0.1141 (3)	0.0211 (10)	
C10	0.4514 (2)	0.8828 (3)	0.1204 (3)	0.0272 (10)	
H10	0.4588	0.9460	0.1170	0.033*	
C11	0.5042 (2)	0.8236 (3)	0.1311 (3)	0.0298 (11)	
H11	0.5477	0.8453	0.1361	0.036*	
C12	0.4922 (2)	0.7305 (3)	0.1347 (3)	0.0295 (11)	
C13	0.4306 (2)	0.6972 (3)	0.1282 (3)	0.0241 (10)	
H13	0.4240	0.6335	0.1299	0.029*	
C14	0.3763 (2)	0.7575 (3)	0.1191 (3)	0.0207 (9)	
C15	0.3135 (2)	0.7189 (3)	0.1168 (3)	0.0202 (9)	
H15	0.3101	0.6546	0.1172	0.024*	
C16	0.1507 (2)	0.7671 (3)	0.1137 (3)	0.0209 (9)	
C17	0.0910 (2)	0.7237 (3)	0.1113 (3)	0.0279 (11)	
H17	0.0889	0.6595	0.1157	0.034*	
C18	0.0352 (2)	0.7776 (3)	0.1022 (3)	0.0315 (11)	
H18	−0.0060	0.7505	0.1005	0.038*	
C19	0.0396 (2)	0.8719 (3)	0.0956 (3)	0.0312 (11)	
H19	0.0016	0.9099	0.0885	0.037*	
C20	0.1007 (2)	0.9079 (3)	0.0996 (3)	0.0269 (10)	
H20	0.1041	0.9718	0.0953	0.032*	
H5	0.214 (2)	0.665 (3)	0.140 (3)	0.022 (13)*	

### Atomic displacement parameters (Å^2^)

**Table:**

	*U*^11^	*U*^22^	*U*^33^	*U*^12^	*U*^13^	*U*^23^
Fe1	0.0230 (3)	0.0106 (3)	0.0143 (3)	0.0020 (3)	0.0071 (3)	−0.0004 (2)
Br1	0.0358 (3)	0.0187 (2)	0.0243 (2)	0.0050 (2)	0.0074 (2)	0.00645 (19)
Br2	0.0304 (3)	0.0502 (4)	0.0369 (3)	0.0172 (2)	0.0073 (2)	−0.0101 (2)
O1	0.0372 (19)	0.0142 (15)	0.0146 (15)	0.0049 (13)	0.0073 (14)	0.0016 (12)
O2	0.0283 (17)	0.0176 (15)	0.0253 (16)	0.0048 (14)	0.0140 (14)	0.0042 (13)
S1	0.0350 (7)	0.0156 (5)	0.0160 (5)	0.0058 (5)	0.0063 (5)	−0.0004 (4)
N1	0.037 (2)	0.019 (2)	0.0144 (18)	0.0031 (17)	0.0049 (17)	0.0010 (15)
N2	0.029 (2)	0.0158 (18)	0.0109 (16)	0.0016 (15)	0.0085 (15)	−0.0026 (14)
N3	0.0220 (19)	0.0145 (18)	0.0129 (17)	0.0002 (15)	0.0082 (15)	−0.0029 (13)
N4	0.0217 (19)	0.0160 (18)	0.0098 (16)	−0.0018 (15)	0.0038 (14)	−0.0010 (13)
N5	0.032 (2)	0.0092 (18)	0.0214 (19)	−0.0001 (16)	0.0096 (17)	0.0003 (15)
N6	0.0219 (19)	0.019 (2)	0.0152 (18)	−0.0012 (16)	0.0035 (15)	−0.0012 (14)
C1	0.024 (2)	0.014 (2)	0.018 (2)	−0.0017 (18)	0.0093 (19)	−0.0021 (16)
C2	0.026 (2)	0.013 (2)	0.020 (2)	0.0003 (18)	0.0116 (19)	−0.0010 (17)
C3	0.022 (2)	0.016 (2)	0.014 (2)	0.0054 (18)	0.0071 (18)	0.0024 (16)
C4	0.033 (3)	0.011 (2)	0.014 (2)	0.0027 (18)	0.0101 (19)	0.0021 (16)
C5	0.020 (2)	0.014 (2)	0.021 (2)	0.0034 (17)	0.0072 (18)	0.0052 (17)
C6	0.030 (3)	0.029 (3)	0.015 (2)	0.004 (2)	0.0070 (19)	0.0036 (19)
C7	0.034 (3)	0.026 (3)	0.012 (2)	0.004 (2)	0.0065 (19)	−0.0018 (18)
C8	0.018 (2)	0.015 (2)	0.018 (2)	0.0013 (17)	0.0056 (18)	0.0027 (16)
C9	0.029 (3)	0.021 (2)	0.014 (2)	0.009 (2)	0.0096 (19)	0.0027 (17)
C10	0.031 (3)	0.029 (3)	0.023 (2)	−0.001 (2)	0.011 (2)	−0.001 (2)
C11	0.023 (3)	0.040 (3)	0.025 (2)	0.000 (2)	0.007 (2)	−0.003 (2)
C12	0.029 (3)	0.035 (3)	0.024 (2)	0.012 (2)	0.009 (2)	−0.004 (2)
C13	0.032 (3)	0.018 (2)	0.022 (2)	0.004 (2)	0.010 (2)	−0.0044 (18)
C14	0.027 (2)	0.023 (2)	0.011 (2)	0.0045 (19)	0.0060 (18)	−0.0031 (17)
C15	0.029 (3)	0.012 (2)	0.013 (2)	0.0022 (18)	0.0000 (18)	−0.0017 (16)
C16	0.030 (2)	0.021 (2)	0.0089 (19)	−0.003 (2)	0.0035 (18)	−0.0010 (16)
C17	0.034 (3)	0.028 (3)	0.024 (2)	−0.008 (2)	0.012 (2)	−0.006 (2)
C18	0.027 (3)	0.035 (3)	0.030 (3)	−0.009 (2)	0.008 (2)	−0.002 (2)
C19	0.026 (3)	0.036 (3)	0.029 (3)	0.004 (2)	0.007 (2)	−0.001 (2)
C20	0.029 (3)	0.027 (2)	0.023 (2)	0.005 (2)	0.009 (2)	−0.002 (2)

### Geometric parameters (Å, º)

**Table:**

Fe1—O2	1.931 (3)	C4—C5	1.359 (5)
Fe1—O1	1.943 (3)	C4—H4	0.9500
Fe1—N4	2.142 (3)	C5—C6	1.396 (6)
Fe1—N6	2.150 (4)	C6—C7	1.362 (6)
Fe1—N3	2.157 (3)	C6—H6	0.9500
Fe1—S1	2.4093 (14)	C7—C8	1.412 (5)
Br1—C5	1.913 (4)	C7—H7	0.9500
Br2—C12	1.921 (4)	C9—C10	1.405 (6)
O1—C8	1.307 (5)	C9—C14	1.418 (6)
O2—C9	1.318 (5)	C10—C11	1.376 (6)
S1—C1	1.720 (4)	C10—H10	0.9500
N1—C1	1.350 (5)	C11—C12	1.399 (6)
N1—H1A	0.8800	C11—H11	0.9500
N1—H1B	0.8800	C12—C13	1.361 (6)
N2—C1	1.314 (5)	C13—C14	1.416 (6)
N2—N3	1.409 (4)	C13—H13	0.9500
N3—C2	1.287 (5)	C14—C15	1.433 (6)
N4—C15	1.298 (5)	C15—H15	0.9500
N4—N5	1.376 (5)	C16—C17	1.403 (6)
N5—C16	1.377 (6)	C17—C18	1.384 (6)
N5—H5	0.83 (4)	C17—H17	0.9500
N6—C20	1.339 (5)	C18—C19	1.400 (6)
N6—C16	1.341 (5)	C18—H18	0.9500
C2—C3	1.454 (5)	C19—C20	1.376 (6)
C2—H2	0.9500	C19—H19	0.9500
C3—C4	1.403 (5)	C20—H20	0.9500
C3—C8	1.416 (5)		
			
O2—Fe1—O1	89.41 (12)	C4—C5—Br1	120.3 (3)
O2—Fe1—N4	84.23 (12)	C6—C5—Br1	119.4 (3)
O1—Fe1—N4	113.13 (12)	C7—C6—C5	119.9 (4)
O2—Fe1—N6	153.25 (13)	C7—C6—H6	120.0
O1—Fe1—N6	85.48 (13)	C5—C6—H6	120.0
N4—Fe1—N6	73.81 (13)	C6—C7—C8	121.7 (4)
O2—Fe1—N3	108.28 (13)	C6—C7—H7	119.2
O1—Fe1—N3	86.13 (12)	C8—C7—H7	119.2
N4—Fe1—N3	157.59 (12)	O1—C8—C7	120.1 (4)
N6—Fe1—N3	97.57 (13)	O1—C8—C3	122.3 (4)
O2—Fe1—S1	97.11 (10)	C7—C8—C3	117.6 (4)
O1—Fe1—S1	165.00 (9)	O2—C9—C10	119.5 (4)
N4—Fe1—S1	81.07 (9)	O2—C9—C14	121.7 (4)
N6—Fe1—S1	94.42 (10)	C10—C9—C14	118.7 (4)
N3—Fe1—S1	79.01 (9)	C11—C10—C9	121.7 (4)
C8—O1—Fe1	135.9 (3)	C11—C10—H10	119.1
C9—O2—Fe1	133.6 (3)	C9—C10—H10	119.1
C1—S1—Fe1	98.35 (14)	C10—C11—C12	118.5 (4)
C1—N1—H1A	120.0	C10—C11—H11	120.8
C1—N1—H1B	120.0	C12—C11—H11	120.8
H1A—N1—H1B	120.0	C13—C12—C11	122.2 (4)
C1—N2—N3	114.1 (3)	C13—C12—Br2	119.1 (4)
C2—N3—N2	112.8 (3)	C11—C12—Br2	118.6 (4)
C2—N3—Fe1	125.0 (3)	C12—C13—C14	119.8 (4)
N2—N3—Fe1	122.2 (2)	C12—C13—H13	120.1
C15—N4—N5	115.8 (4)	C14—C13—H13	120.1
C15—N4—Fe1	127.5 (3)	C13—C14—C9	119.0 (4)
N5—N4—Fe1	116.1 (2)	C13—C14—C15	117.4 (4)
N4—N5—C16	115.6 (4)	C9—C14—C15	123.5 (4)
N4—N5—H5	118 (3)	N4—C15—C14	124.2 (4)
C16—N5—H5	122 (3)	N4—C15—H15	117.9
C20—N6—C16	118.2 (4)	C14—C15—H15	117.9
C20—N6—Fe1	125.1 (3)	N6—C16—N5	116.9 (4)
C16—N6—Fe1	116.6 (3)	N6—C16—C17	122.8 (4)
N2—C1—N1	116.6 (4)	N5—C16—C17	120.3 (4)
N2—C1—S1	126.4 (3)	C18—C17—C16	117.6 (4)
N1—C1—S1	117.0 (3)	C18—C17—H17	121.2
N3—C2—C3	127.3 (4)	C16—C17—H17	121.2
N3—C2—H2	116.4	C17—C18—C19	120.0 (4)
C3—C2—H2	116.4	C17—C18—H18	120.0
C4—C3—C8	119.5 (4)	C19—C18—H18	120.0
C4—C3—C2	117.5 (4)	C20—C19—C18	117.8 (4)
C8—C3—C2	123.0 (4)	C20—C19—H19	121.1
C5—C4—C3	121.0 (4)	C18—C19—H19	121.1
C5—C4—H4	119.5	N6—C20—C19	123.6 (5)
C3—C4—H4	119.5	N6—C20—H20	118.2
C4—C5—C6	120.3 (4)	C19—C20—H20	118.2

### Hydrogen-bond geometry (Å, º)

**Table:**

*D*—H···*A*	*D*—H	H···*A*	*D*···*A*	*D*—H···*A*
N5—H5···N2^i^	0.83 (4)	2.00 (4)	2.825 (5)	171 (4)
N1—H1*A*···O2^ii^	0.88	2.29	2.987 (4)	136

Symmetry codes: (i) −*x*+1/2, *y*−1/2, −*z*+1/2; (ii) *x*, −*y*+2, *z*+1/2.
